# Association of cord blood vitamin D and genetic polymorphisms with childhood food allergy in Shanghai, China: a prospective cohort

**DOI:** 10.3389/fnut.2025.1652487

**Published:** 2025-08-25

**Authors:** Xirui Wang, Yingying Cai, Jingjing Pei, Bin Wang, Ying Tian, Jun Zhang, Xiaodan Yu

**Affiliations:** ^1^Department of Developmental and Behavioral Pediatrics, Shanghai Children’s Medical Center, Shanghai Jiao Tong University School of Medicine, Pudong, Shanghai, China; ^2^Developmental and Behavior Pediatrics Department, Fujian Branch of Shanghai Children’s Medical Center, Fujian Children’s Hospital, Fuzhou, China; ^3^Nutrition Department, Wuxi Maternal and Child Health Care Hospital, Women’s Hospital of Jiangnan University, Jiangnan University, Wuxi, China; ^4^MOE-Shanghai Key Laboratory of Children’s Environmental Health, Xinhua Hospital, Shanghai Jiao Tong University School of Medicine, Yangpu, Shanghai, China

**Keywords:** vitamin D, genetic polymorphisms, food allergy, birth cohort, gene–environment interaction

## Abstract

**Background:**

Emerging evidence suggests vitamin D plays a dual role in immune regulation, yet its interplay with genetic susceptibility in early-life allergy development remains poorly understood. This prospective cohort study investigated whether cord blood 25-hydroxyvitamin D [25(OH)D] levels interact with immunoregulatory gene variants to influence childhood food allergy risk.

**Methods:**

A total of 1,049 mother-infant pairs from the Shanghai Allergy Cohort were stratified by cord blood 25(OH)D concentrations (<15, 15–25, >25 ng/mL). Food allergy diagnoses at 6, 12, and 24 months followed standardized clinical criteria. Five single-nucleotide polymorphisms (SNPs) (IL4, IL4R, IL13, MS4A2) were genotyped using MALDI-TOF MS. Multivariable logistic regression evaluated associations between vitamin D, genetic polymorphisms, and allergy outcomes, adjusting for birth season, maternal allergy history, and environmental confounders. Gene-vitamin D interactions were tested via stratified analyses.

**Results:**

A U-shaped relationship was observed between cord blood serum25(OH)D levels and the risk of developing childhood food allergies. Both deficient (<15 ng/mL) and elevated (>25 ng/mL) 25(OH)D levels at birth independently increased 6-month food allergy risk (adjusted OR = 2.55 and 2.38, respectively). By 24 months, only deficient levels showed attenuated effects (OR = 1.14, *p* = 0.779). IL4R rs1801275 AA, IL13 rs20541 GG, and IL-4 rs2243250 CC genotypes synergistically amplified allergy risk under vitamin D deficiency (adjusted OR = 26.14, *p* = 0.019; OR = 6.51, *p* = 0.025; OR = 4.13, *p* = 0.007). Notably, the protective effect of MS4A2 rs569108 GG genotype observed at reference vitamin D levels (adjusted OR = 0.55, *p* = 0.016) was attenuated at high levels (OR = 0.68, *p* = 0.149).

**Conclusion:**

Genetic susceptibility in Th2 pathway genes (IL4R, IL-4, IL13) dramatically amplified food allergy risk under vitamin D deficiency, with AA/GG/CC genotypes conferring 4- to 26-fold increased susceptibility. Conversely, the protective effect of MS4A2 rs569108 GG genotype was compromised at high vitamin D levels (>25 ng/mL). Our findings underscore that personalized vitamin D thresholds during pregnancy must account for fetal genetic background to mitigate allergy risk.

## 1 Introduction

While the effects of vitamin D deficiency on bone health are well documented, its role in other health outcomes is a contentious issue in many areas of medicine, including allergy and immunology. Vitamin D is increasingly recognized for its significant role as an immunomodulatory agent, influencing various aspects of the immune system, including allergies and immunology (1, 2). Interest in the role of vitamin D in the incidence of allergic diseases has significantly increased in recent years, largely due to the perception of vitamin D supplementation as an affordable, noninvasive, and easily administered intervention. Research has shown that vitamin D deficiency is often associated with an increased risk of developing allergic diseases. For instance, children with lower vitamin D levels have been found to have a higher likelihood of food allergies, particularly in their early years (3). This association is thought to be due to vitamin D’s role in maintaining the integrity of the epithelial barrier and promoting a more tolerogenic immune response, which can prevent the immune system from overreacting to harmless antigens (4). However, the evidence regarding the impact of vitamin D on allergy-related outcomes remains inconclusive.

The proposed mechanism by which vitamin D influences immune development and immune-related diseases involves both direct and indirect effects on the immune system. In their review of the impacts of gut microbiota, probiotics, and vitamin D on allergies and asthma, Ly et al. posited that vitamin D may serve as a significant modifier in the relationship between intestinal flora and inflammatory disorders (5). Recent discussions have suggested that low levels of vitamin D may constitute a risk factor for inflammatory bowel diseases, such as Crohn’s disease, indicating that vitamin D can influence gut inflammation (6, 7). This gut inflammation, in turn, may impact immune development and function, potentially manifesting as the initial observable inflammatory diseases, such as eczema and allergies (8). The conversion of 25-hydroxyvitamin D [25(OH)D] to its active form, 1,25-dihydroxyvitamin D [1,25(OH)2D], within monocytes and macrophages enhances the synthesis of antimicrobial peptides, thereby augmenting cellular immune responses. Vitamin D inhibits the proinflammatory responses of the adaptive immune system, and promotes proliferation of immunosuppressive regulatory T cells and their accumulation at sites of inflammation (9, 10).

Given the promising data from clinical trials, the inconsistent findings from observational studies—including the German LINA cohort where higher maternal vitamin D correlated with increased food allergy risk (11)—regarding intrauterine vitamin D status and atopic disease incidence remain perplexing (12–15). The significant clustering of allergies within families indicates a genetic foundation, with several genetic polymorphisms being involved (16–19). Liu et al. proposed that vitamin D might interact with or modify genetic predispositions in children with high-risk genotypes, based on their findings of a significant interaction between an IL-4 gene polymorphism and vitamin D deficiency (20). This interaction was associated with an increased risk of food sensitization among children with CC/CT genotypes (OR = 1.79, 95% CI: 1.15–2.77). To comprehensively understand the relationship between intrauterine vitamin D exposure and allergic diseases, it is essential to conduct analyses using well-designed, large-scale prospective cohorts of maternal–infant dyads. These studies should account for individual genetic risk, early life exposures, and environmental confounders. This study aims to assess the relationship between maternal and cord blood vitamin D status and atopic outcomes within a thoroughly characterized prospective birth cohort, featuring clinically validated outcomes and long-term follow-up.

The influence of early-life vitamin D on the development of childhood allergies remains a subject of debate. Polymorphisms in genes are hypothesized to modulate the effects of vitamin D *in vivo*. This study aims to investigate the associations between vitamin D exposure and genetic polymorphisms in the context of childhood food allergy development.

## 2 Methods

### 2.1 Study population

The data we consider arises from a prospective birth cohort study in Shanghai, called the Shanghai Allergy Cohort Study (21). Between 2012 and 2013, 1,269 mother-infant pairs were enrolled at two major tertiary hospitals in Shanghai: Xinhua Hospital and the International Peace Maternity and Child Hospital. Written informed consent was secured from the mothers before delivery, and face-to-face interviews were conducted by trained nurses. Upon birth, the study nurses obtained the newborn’s anthropometric data and, when accessible, umbilical cord blood. Ethics approval was obtained by the Ethics Committees of both Xinhua Hospital affiliated to Shanghai Jiao Tong University School of Medicine and the International Maternal and Children Care Hospital (approval number: XHEC-C-2012-003) and conducted according to the principles in the Declaration of Helsinki.

### 2.2 Clinical assessment and sample collection

Trained research reviewers conducted face-to-face interviews using structured questionnaires, collecting information on maternal age, height, prepregnancy weight, education level, socioeconomic status, maternal atopy, prenatal pet exposure, prenatal active or secondhand smoking, vitamin D and other multi-vitamin supplement intake, outdoor activity during pregnancy, and smoking status of the mother, father, coworkers and relatives. Maternal atopy was referred to those mothers who had asthma, allergic rhinitis or atopic dermatitis along with detectable specific IgE. Prenatal pet exposure was defined as keeping cats or dogs at home during pregnancy. Information on parity, previous pregnancy, gestational age, date of birth, delivery mode, infants’ gender, birth weight and antenatal complications was obtained from medical records.

Interviewer-led questionnaires and clinical assessments were conducted at 6, 12 and 24 months. Data on infant feeding, detailing breast-feeding, formula or combination feeding, frequency of feeds, name and brand of infant formula, complementary feeding, eating behavior, and supplementation was collected at each time-point. Anthropometric measures of weight, length, and abdominal circumference were measured using standard operating procedures. Longitudinal screening to assess suspected food allergy was performed at each clinical assessment visit using clinical diagnostic criteria.

### 2.3 Measurements of umbilical cord blood 25(OH)D

As described before (21), the data contains exposures to 25(OH)D were measured in cord serum as the primary marker of maternal vitamin D exposure. The primary measurement of the present study was the cord serum level of 25(OH)D. Cord blood was available for 1,071 of the newborn participants. Maternal blood during pregnancy or postnatal infant vitamin D measurements were not obtained due to ethical and practical constraints associated with repeated blood sampling in this cohort. The samples of cord blood were spun and placed in −80°C freezers within a two-hour window. We utilized the sensitive LC–MS/MS method to analyze serum 25(OH)D as per the procedure reported by our previous study (22). In this assay, the level of sensitivity for LC/MS/MS assay was 0.05 ng/mL for 25(OH)D_2_, and 0.1 ng/mL for 25(OH)D_3_. The serum samples (100 μL) underwent deproteinization and precipitation with methanol, acetonitrile, zinc sulfate, and internal standards, including deuterated 25(OH)D_2_ and 25(OH)D_3_ (Sigma, St. Louis, MO, USA). Chromatographic separations were achieved using a 50 × 2.1 mm, 2.7 μm Agilent Poroshell 120 EC-C18 column, with a mobile phase gradient of water containing 0.1% formic acid and methanol, at a flow rate of 0.5 mL/min. Multiple reaction monitoring (MRM) of the analyses was performed under electrospray ionization (ESI) in the positive mode at *m/z* 401.3 → 383.2 and 401.3 → 159.1 for 25(OH)D_3_, *m/z* 413.3 → 395.3 and 413.3 → 355.2 for 25(OH)D_2_, and *m/z* 404.3 → 386.3 and 416.4.3 → 398.3 for d3-25(OH)D_3_ and d3-25(OH)D_2_, respectively. Although there is no consensus on optimal levels of 25(OH)D as measured in cord blood serum (23, 24), vitamin D level has been divided into three groups [25(OH)D <15 ng/mL; 15–25 ng/mL; >25 ng/mL] according to our previous study (25).

### 2.4 Selection of single nucleotide polymorphisms and genotyping

This investigation targeted four candidate genes, including IL13, IL4, IL4RA, and FCER1B, which are significant inflammatory genes impacting IgE levels and have been associated with atopy in more than ten studies (26–28). An earlier study recognized gene interactions linked to asthma in Chinese Han children (29). Five functional single-nucleotide polymorphisms (SNPs) with a minor allele frequency exceeding 10% were selected for analysis from these genes (30).

The QIAamp DNA Blood Mini Kit (QIAGEN, Hilden, Germany) was used to extract genomic DNA from cord blood. Genotyping of the five SNPs was conducted using MALDI-TOF MS, which stands for matrix-assisted laser desorption/ionization time-of-flight mass spectrometry (30), using the MassARRAY iPLEX platform (Sequenom Inc., San Diego, CA, USA) according to the manufacturer’s instructions. The overall call rate was 98.6%. The quality control for genotyping involved 5% duplicate and negative samples, with a concordance rate exceeding 98%.

### 2.5 Statistical analysis

Data was analyzed using a prospective cohort design to evaluate associations between cord blood vitamin D levels, genetic polymorphisms, and childhood food allergy. Continuous variables were expressed as mean ± standard deviation (SD) and compared across vitamin D strata (<15, 15–25, >25 ng/mL) using one-way ANOVA or non-parametric Kruskal-Wallis tests, as appropriate. Categorical variables were summarized as counts (%) and analyzed via CMH- χ^2^ or Fisher’s exact tests. The deviations from Hardy–Weinberg equilibrium (HWE) were tested using χ^2^ tests to ensure genotype frequencies in the study population conformed to expected distributions. Polymorphisms violating HWE (*p* < 0.05) were flagged for sensitivity analyses. Univariate and multivariate logistic regression models were employed to estimate odds ratios (ORs) and 95% confidence intervals (CIs) for associations between vitamin D levels and food allergy at 6, 12, and 24 months. Multivariate models adjusted for covariates identified according to the clinical experience, including season of birth, newborn sex, maternal allergy history, household pet ownership, pre-pregnancy smoking, antibiotic use during pregnancy, birth weight, APGAR score, maternal BMI, and maternal age. Sensitivity analyses confirmed model robustness. Genetic polymorphisms were evaluated under dominant and recessive inheritance models. Interaction effects between vitamin D levels and genotypes were tested using stratified logistic regression, with multiplicative interaction terms. Adjusted ORs were reported after controlling for confounding variables. A two-tailed *p*-value <0.05 defined statistical significance. Sensitivity analyses treated cord blood 25(OH)D as a continuous variable using restricted cubic splines with three knots (10th, 50th, 90th percentiles) to model non-linear associations and test dose–response relationships. Gene–environment interactions were formally tested by including multiplicative interaction terms (genetic model × vitamin D category) in adjusted logistic regression models. Dominant/recessive models were selected based on prior functional evidence (31). Given the non-linear relationship between continuous 25(OH)D and allergy risk (see Results 3.2), genotype-stratified analyses are presented using categorical vitamin D. All analyses were performed using SAS v9.4 (SAS Institute), with missing data addressed via complete-case analysis, as missingness was <5% for all variables. Results were reported in accordance with STROBE guidelines to ensure methodological transparency.

## 3 Results

### 3.1 Baseline characteristics of the cohort population

**Figure 1:**
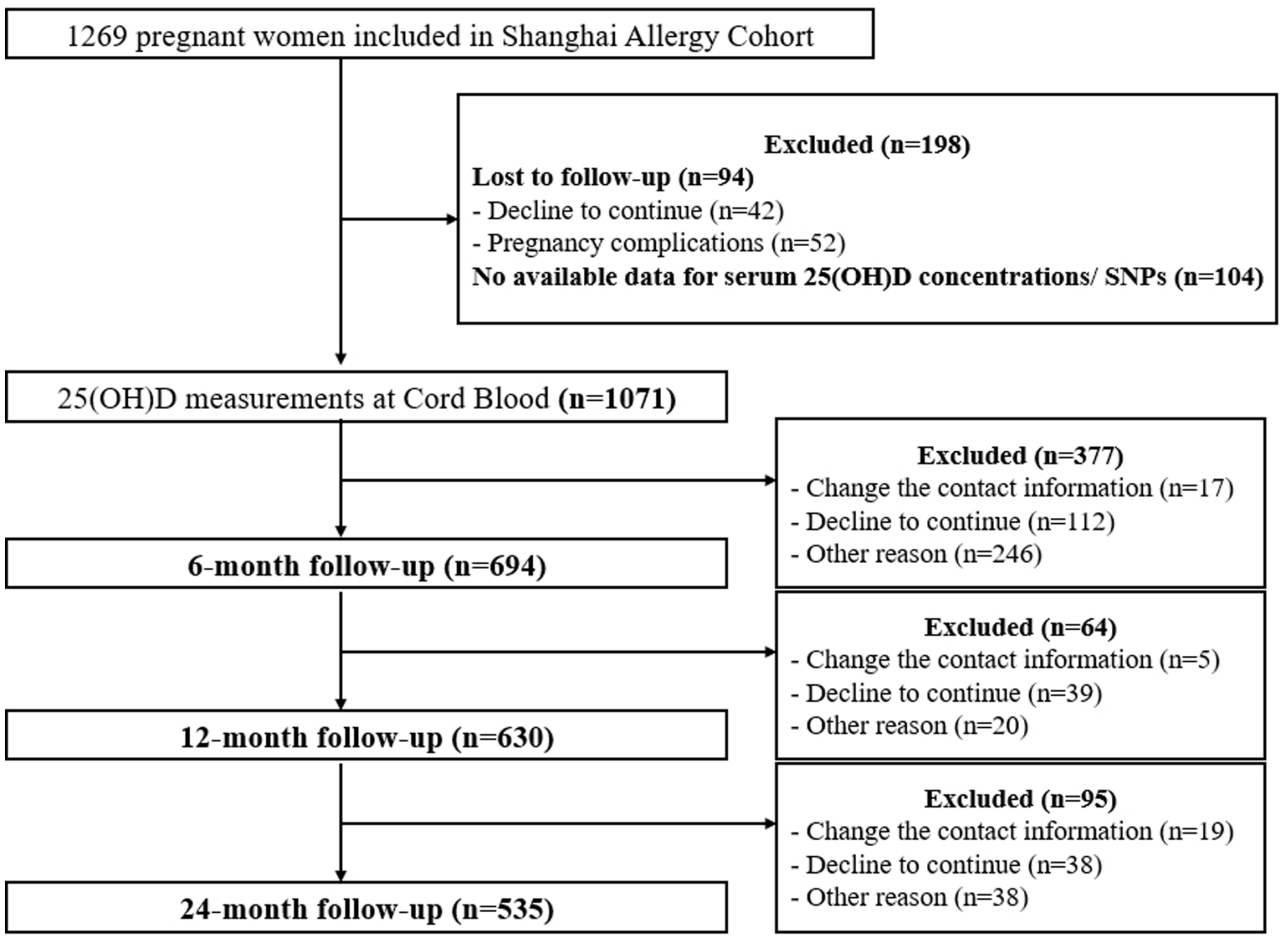
Flow chart of the study protocol. 25(OH)D, 25-hydroxyvitamin D; SNPs, single-nucleotide polymorphisms.

The flow chart of the study protocol is shown in Figure 1. Effective cord blood was available for 1,049 of the newborn participants. None of the participants were included twice during the study. General characteristics of the pregnant women and their infants are present in Table 1. The study cohort comprised 1,049 mother–child pairs stratified by cord blood vitamin D levels: <15 ng/mL (*N* = 37), 15–25 ng/mL (*N* = 611), and >25 ng/mL (*N* = 401). Significant differences were observed across vitamin D groups for paternal age (mean ± SD: 30.94 ± 3.98 vs. 31.38 ± 4.29 vs. 32.10 ± 4.94; *p* = 0.037), gravidity (*p* = 0.0028), gestational age categories (*p* = 0.0028), and season of birth (*p* < 0.001). Infants born between April and June had the lowest vitamin D levels, while those born between October and December had the highest (*p* < 0.001). No significant differences were found in maternal age, BMI, birth weight, race/ethnicity, mode of delivery, sex, maternal/paternal allergy history, household income, education levels, pet ownership, smoking exposure, or pregnancy complications (e.g., preeclampsia, gestational diabetes mellitus). However, infants in the lowest vitamin D group (<15 ng/mL) showed a higher proportion of 6-month-old food allergy (11.11% vs. 4.46% in the reference group [15–25 ng/mL], *p* = 0.0067), persisting to 24 months (42.86% vs. 19.49%, *p* = 0.014).

**Table 1:** Baseline characteristics of the cohort population and descriptive analysis of groups stratified by vitamin D levels.

Characteristics		Vitamin D levels			Statistics	*p* value
		<15 (*N* = 37)	15 ~ 25 (*N* = 611)	>25 (*N* = 401)		
Maternal age (year)	Mean (SD)	28.94 (3.31)	29.05 (3.38)	29.41 (3.84)	1.31	0.272
Paternal age (year)	Mean (SD)	30.94 (3.98)	31.38 (4.29)	32.10 (4.94)	3.3	**0.037**
Maternal BMI (kg/m^2^)	Mean (SD)	21.94 (3.51)	21.44 (3.67)	21.37 (3.21)	0.44	0.646
Gestational age (week)	Mean (SD)	38.78 (1.17)	38.82 (1.32)	38.65 (1.28)	2.13	0.119
Birth weight (g)	Mean (SD)	3475.97 (433.51)	3413.67 (466.79)	3369.80 (459.44)	1.58	0.206
Race/Ethnicity	Han Chinese	34 (91.89)	579 (94.76)	385 (96.01)	1.69	0.430
	Other	3 (8.11)	32 (5.24)	16 (3.99)		
Parity	0	33 (91.67)	544 (91.43)	345 (87.12)	7.18	0.127
	1	3 (8.33)	49 (8.24)	51 (12.88)		
	2	0 (0.00)	2 (0.34)	0 (0.00)		
Gravidity	0	24 (66.67)	393 (66.05)	257 (64.90)	16.17	**0.003**
	1	11 (30.56)	149 (25.04)	77 (19.44)		
	>1	1 (2.78)	53 (8.91)	62 (15.66)		
Gestational age	<38	3 (8.33)	53 (8.91)	39 (9.85)	8.57	0.199
	38	14 (38.89)	160 (26.89)	125 (31.57)		
	39	9 (25.00)	218 (36.64)	148 (37.37)		
	>39	10 (27.78)	164 (27.56)	84 (21.21)		
Season of birth	April through June	10 (29.41)	112 (18.95)	60 (15.31)	37.37	**<0.001**
	July through September	20 (58.82)	297 (50.25)	147 (37.50)		
	October through December	4 (11.76)	182 (30.80)	185 (47.19)		
Mode of delivery	Cesarean birth	8 (22.22)	134 (22.52)	97 (24.49)	0.54	0.763
	Natural birth	28 (77.78)	461 (77.48)	299 (75.51)		
Sex of the newborn	Male	18 (50.00)	318 (53.45)	198 (50.13)	1.11	0.574
	Female	18 (50.00)	277 (46.55)	197 (49.87)		
Maternal allergy	No	26 (78.79)	517 (87.04)	332 (86.01)	1.88	0.391
	Yes	7 (21.21)	77 (12.96)	54 (13.99)		
Paternal allergy	No	0 (0.00)	7 (11.48)	6 (14.29)	0.76	0.684
	Yes	4 (100.00)	54 (88.52)	36 (85.71)		
Household income (RMB/yr)	<200,000	12 (44.44)	160 (32.26)	92 (29.68)	5.98	0.201
	200,000–500,000	12 (44.44)	221 (44.56)	131 (42.26)		
	>500,000	3 (11.11)	115 (23.19)	87 (28.06)		
Maternal education level	High school degree or below	8 (22.86)	83 (13.93)	56 (14.18)	2.77	0.597
	College degree	25 (71.43)	464 (77.85)	302 (76.46)		
	Bachelor degree or above	2 (5.71)	49 (8.22)	37 (9.37)		
Paternal education level	High school degree or below	6 (17.14)	82 (13.78)	50 (12.76)	2.08	0.720
	College degree	27 (77.14)	443 (74.45)	291 (74.23)		
	Bachelor degree or above	2 (5.71)	70 (11.76)	51 (13.01)		
Household pet ownership	No	33 (94.29)	539 (90.59)	340 (86.51)	5.05	0.080
	Yes	2 (5.71)	56 (9.41)	53 (13.49)		
Pre-pregnancy smoking	No	35 (100.00)	592 (99.16)	385 (97.72)	4.19	0.123
	Yes	0 (0.00)	5 (0.84)	9 (2.28)		
*In utero* smoking exposure	No	15 (40.54)	328 (53.68)	221 (55.11)	2.89	0.235
	Yes	22 (59.46)	283 (46.32)	180 (44.89)		
Prenatal complications	No	27 (77.14)	485 (83.33)	299 (78.27)	4.25	0.119
	Yes	8 (22.86)	97 (16.67)	83 (21.73)		
Preeclampsia	No	35 (97.22)	561 (95.41)	363 (94.29)	0.99	0.607
	Yes	1 (2.78)	27 (4.59)	22 (5.71)		
GDM	No	32 (88.89)	533 (90.49)	332 (86.01)	4.68	0.096
	Yes	4 (11.11)	56 (9.51)	54 (13.99)		
Antibiotic use during pregnancy	No	32 (91.43)	574 (96.31)	381 (96.95)	2.84	0.242
	Yes	3 (8.57)	22 (3.69)	12 (3.05)		
Gestational glucose intolerance	No	36 (100.00)	582 (99.32)	389 (99.49)		
	Yes	0 (0.00)	4 (0.68)	2 (0.51)		
6-month-old food allergy	No	24 (88.89)	386 (95.54)	235 (89.35)	10.01	**0.0067**
	Yes	3 (11.11)	18 (4.46)	28 (10.65)		
12-month-old food allergy	No	21 (84.00)	342 (91.44)	200 (86.58)	4.34	0.114
	Yes	4 (16.00)	32 (8.56)	31 (13.42)		
24-month-old food allergy	No	12 (57.14)	252 (80.51)	147 (73.13)	8.50	**0.014**
	Yes	9 (42.86)	61 (19.49)	54 (26.87)		

Bold values: *p* < 0.05.

### 3.2 Associations between vitamin D levels and food allergy

**Figure 2:**
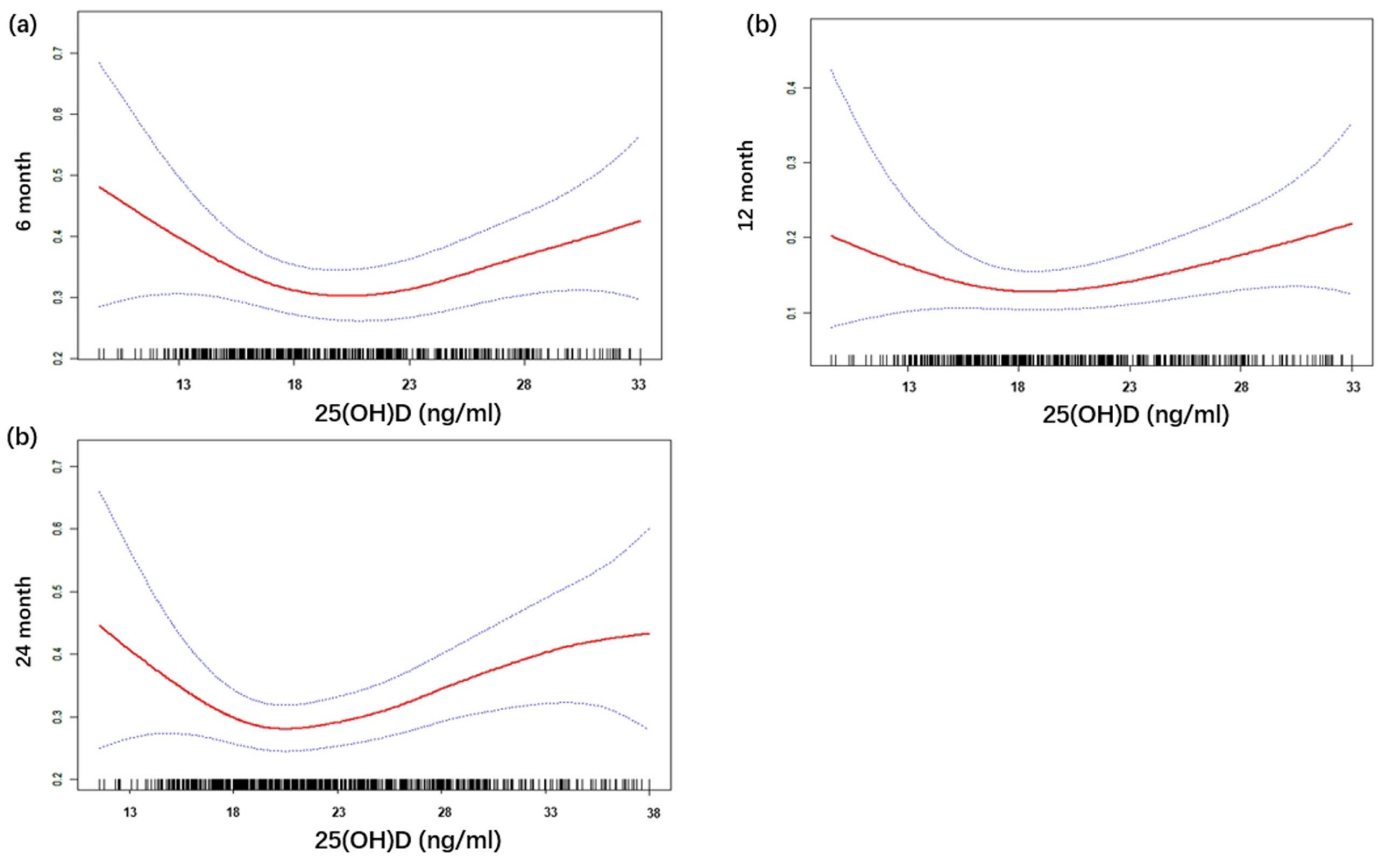
Relationship between cord blood vitamin D and food allergy status at: **(a)** 6-month; **(b)** 12-month; **(c)** 24-month. 25(OH)D, 25-hydroxyvitamin D.

In univariate analysis, both low (<15 ng/mL) and high (>25 ng/mL) vitamin D levels were associated with increased risk of food allergy at 6 months (OR = 2.40, 95% CI: 1.18–4.90, *p* = 0.016; OR = 2.56, 95% CI: 1.38–4.72, *p* = 0.003) and 12 months (OR = 2.08, 95% CI: 1.35–3.19, *p* = 0.001; OR = 1.62, 95% CI: 1.12–2.36, *p* = 0.011) (Supplementary Table S1). Restricted cubic spline curves model confirmed a significant U-shaped dose–response relationship (Figure 2). Risk increased by 18–22% per 5 ng/mL decrease below 20 ng/mL and 10–18% per 5 ng/mL increase above 25 ng/mL at 6–12 months (all *p* < 0.05). After adjusting for covariates (season of birth, sex, maternal allergy, pet ownership, smoking, antibiotic use, birth weight, APGAR score, maternal BMI, and age), these associations remained significant for 6-month (adjusted OR = 2.55, *p* = 0.015 for <15 ng/mL; adjusted OR = 2.38, *p* = 0.008 for >25 ng/mL) and 12-month allergies (adjusted OR = 1.98, *p* = 0.035 for <15 ng/mL) (Table 2). By 24 months, only low vitamin D retained marginal significance in univariate analysis (OR = 1.55, *p* = 0.027), but not after adjustment (adjusted OR = 1.14, *p* = 0.779) (Table 2). For cumulative 0–24 month allergy risk, high vitamin D (>25 ng/mL) showed a robust association (adjusted OR = 1.78, 95% CI: 1.19–2.67, *p* = 0.005), while low vitamin D trended toward significance (adjusted OR = 1.50, *p* = 0.044) (Table 2).

**Table 2:** Associations between vitamin D levels and food allergy: categorical and continuous vitamin D model.

Cord blood 25(OH)D model	6-month aOR (95% CI)	12-month aOR (95% CI)	24-month aOR (95% CI)
Categorical: 15 ~ 25 ng/mL	Ref.	Ref.	Ref.
Categorical: <15 ng/mL	**2.55 (1.20–5.43)**	**1.98 (1.05–3.73)**	1.14 (0.45–2.89)
Categorical: >25 ng/mL	**2.38 (1.26–4.52)**	1.46 (0.84–2.54)	1.26 (0.62–2.57)
Continuous (per 5 ng/mL): 15 ~ 25 ng/mL	Ref.	Ref.	Ref.
Continuous (per 5 ng/mL): <20 ng/mL	**1.22 (1.06–1.40)**	**1.15 (1.02–1.30)**	1.01 (0.87–1.18)
Continuous (per 5 ng/mL): >25 ng/mL	**1.18 (1.04–1.34)**	**1.10 (1.00.–1.22)**	1.04 (0.91–1.19)
*p* for non-linearity	<0.001	<0.001	<0.001

Covariates adjusted for in multivariate analysis included season of birth, sex of the newborn, maternal allergy, household pet ownership, pre-pregnancy smoking, antibiotic use during pregnancy, birth weight, APGAR score, maternal BMI, maternal age.

Bold values: *p* < 0.05.

### 3.3 Genetic polymorphisms and food allergy susceptibility

Among five genetic loci analyzed, rs569108 (MS4A2 gene) demonstrated a significant association with food allergy under the dominant model (GG vs. AA+AG: adjusted OR = 1.68, 95% CI: 1.13–2.49, *p* = 0.011). No significant associations were observed for rs2243250 (IL-4), rs1801275 (IL-4R), rs1295686 (IL13), or rs20541 (IL13) in either dominant or recessive models after covariate adjustment. However, rs2243250 (IL-4) exhibited a trend toward reduced allergy risk in the dominant model (TT vs. TC+CC: adjusted OR = 0.59, 95% CI: 0.34–1.03, *p* = 0.062) (Table 3).

**Table 3:** Analysis of 0–24 month food allergy susceptibility based on the genotype and locus genetic model.

Food allergy			Univariate analysis		Multivariate analysis	
SNPs	Genotype/Genetic model		OR (95%CI)	*p* value	OR (95%CI)	*p* value
rs2243250 (IL-4 gene)	CC		Ref.		Ref.	
	TC		1.02 (0.33, 3.16)	0.464	0.87 (0.27, 2.78)	0.62
	TT		0.61 (0.20, 1.87)	0.146	0.52 (0.17, 1.64)	0.103
	Dominant model	TC+CC	Ref.		Ref.	
		TT	0.61 (0.36, 1.02)	0.06	0.59 (0.34, 1.03)	0.062
	Recessive model	CC	Ref.		Ref.	
		TT+TC	0.74 (0.25, 2.20)	0.589	0.64 (0.21, 1.95)	0.43
rs1801275 (IL_4R gene)	AA		Ref.		Ref.	
	AG		0.89 (0.57, 1.39)	0.551	0.78 (0.49, 1.26)	0.759
	GG		0.48 (0.11, 2.17)	0.376	0.47 (0.10, 2.18)	0.415
	Dominant model	AA+AG	Ref.		Ref.	
		GG	0.50 (0.11, 2.24)	0.362	0.50 (0.11, 2.33)	0.38
	Recessive model	AA	Ref.		Ref.	
		GG+AG	0.86 (0.55, 1.32)	0.482	0.75 (0.47, 1.20)	0.231
	CC		Ref.		Ref.	
	TC		1.20 (0.58, 2.49)	0.343	1.41 (0.62, 3.19)	0.215
	TT		0.91 (0.43, 1.91)	0.45	1.03 (0.45, 2.36)	0.608
rs1295686 (IL13 gene)	Dominant model	CC	Ref.		Ref.	
		TT vs. TC	0.78 (0.51, 1.19)	0.246	0.77 (0.50, 1.21)	0.257
	Recessive model	CC	Ref.		Ref.	
		TT+TC	1.05 (0.52, 2.12)	0.893	1.21 (0.55, 2.67)	0.628
	AA		Ref.		Ref.	
	AG		1.37 (0.89, 2.12)	0.189	1.43 (0.90, 2.27)	0.096
	GG		0.99 (0.46, 2.12)	0.645	0.83 (0.35, 1.96)	0.387
rs20541 (IL13 gene)	Dominant model	AA+AG	Ref.		Ref.	
		GG	0.83 (0.40, 1.72)	0.623	0.69 (0.30, 1.56)	0.374
	Recessive model	AA	Ref.		Ref.	
		GG+AG	1.30 (0.85, 1.98)	0.22	1.32 (0.84, 2.06)	0.224
	AA		Ref.		Ref.	
	AG		0.62 (0.21, 1.80)	0.128	0.58 (0.19, 1.72)	0.085
	GG		1.01 (0.36, 2.82)	0.401	1.03 (0.36, 2.93)	0.315
rs569108 (MS4A2 gene)	Dominant model	AA+AG	Ref.		Ref.	
		GG	**1.55 (1.06, 2.27)**	**0.024**	**1.68 (1.13, 2.49)**	**0.011**
	Recessive model	AA	Ref.		Ref.	
		GG+AG	0.88 (0.32, 2.46)	0.808	0.88 (0.31, 2.50)	0.813

Covariates adjusted for in multivariate analysis included season of birth, sex of the newborn, maternal allergy, household pet ownership, pre-pregnancy smoking, antibiotic use during pregnancy, birth weight, APGAR score, maternal BMI, maternal age.

Bold values: *p* < 0.05.

### 3.4 Gene-vitamin D interaction effects

Stratified analyses revealed interactions between vitamin D levels and specific polymorphisms. For rs1801275 (IL-4R), infants with the AA genotype and low vitamin D (<15 ng/mL) had a significantly elevated allergy risk (adjusted OR = 26.14, 95% CI: 2.56–267.11, *p* = 0.019). Similarly, rs20541 (IL13) GG genotype infants with low vitamin D showed significantly elevated risk (adjusted OR = 6.51, 95% CI: 1.30–32.60, *p* = 0.025). Additionally, rs2243250 (IL-4) CC homozygotes with vitamin D deficiency exhibited elevated allergy risk (adjusted OR = 4.13, 95% CI: 1.48–11.57, *p* = 0.007). Notably, in the reference vitamin D group (15–25 ng/mL), the rs569108 (MS4A2) GG genotype conferred protection against allergy (adjusted OR = 0.55, 95% CI: 0.32–0.94, *p* = 0.016), but this effect was attenuated at high vitamin D levels (>25 ng/mL; adjusted OR = 0.68, 95% CI: 0.37–1.25, *p* = 0.149) (Table 4). The results of stratified analyses by genotype for all SNPs, evaluating both categorical and continuous vitamin D are comprehensively detailed in Supplementary Table S3. These interactions demonstrate that genetic susceptibility in Th2 pathway genes (IL4R, IL-4, IL13) dramatically amplifies food allergy risk under vitamin D deficiency, while MS4A2 variants modulate vitamin D’s protective effects.

**Table 4:** Analysis of the interaction of vitamin D and gene polymorphism on 0–24 month food allergy.

SNPs	Genetic model		25(OH)D level	Univariate analysis		Multivariate analysis		Significant interaction
				OR (95%CI)	*p* value	OR (95%CI)	*p* value	
rs2243250 (IL-4 gene)	Dominant model	TC+CC	15 ~ 25	Ref.	–	Ref.	–	–
		TC+CC	<15	2.91 (0.88, 9.69)	0.218	3.34 (0.96, 11.65)	0.058	No
		TC+CC	>25	1.40 (0.82, 2.41)	0.216	1.28 (0.72, 2.28)	0.404	No
		TT	15 ~ 25	1.08 (0.60, 1.95)	0.785	1.00 (0.54, 1.86)	0.856	No
		TT	<15	3.26 (0.84, 12.74)	0.089	4.30 (0.84, 21.87)	0.083	No
		TT	>25	1.47 (0.77, 2.80)	0.246	1.29 (0.64, 2.60)	0.477	No
	Recessive model	CC	15 ~ 25	Ref.	–	Ref.	–	–
		**CC**	**<15**	**2.92 (1.12, 7.61)**	**0.028**	**4.13 (1.48, 11.57)**	**0.007**	**Yes**
		CC	>25	1.41 (0.91, 2.19)	0.126	1.26 (0.79, 2.02)	0.341	No
		TT+TC	15 ~ 25	1.24 (0.39, 3.94)	0.723	1.11 (0.33, 3.73)	0.866	No
		TT+TC	<15	4.02 (0.25, 65.23)	0.322	3.64 (0.45, 32.47)	0.482	No
		TT+TC	>25	1.34 (0.26, 6.81)	0.730	2.69 (0.47, 15.49)	0.265	No
rs1801275 (IL_4R gene)	Dominant model	AA+AG	15 ~ 25	Ref.	–	Ref.	–	–
		AA+AG	>25	0.19 (0.01, 2.50)	0.212	0.13 (0.01, 1.76)	0.123	No
		GG	15 ~ 25	0.75 (0.15, 3.83)	0.731	0.59 (0.11, 3.15)	0.538	No
		GG	<15	2.25 (0.37, 13.87)	0.380	2.15 (0.32, 14.52)	0.431	No
		GG	>25	1.11 (0.22, 5.70)	0.899	0.83 (0.15, 4.48)	0.832	No
	Recessive model	AA	15 ~ 25	Ref.	–	Ref.	–	–
		**AA**	**<15**	**25.28 (2.78, 229.45)**	**0.011**	**26.14 (2.56, 267.11)**	**0.019**	**Yes**
		AA	>25	1.62 (0.76, 3.45)	0.208	1.57 (0.71, 3.50)	0.266	No
		GG+AG	15 ~ 25	1.45 (0.79, 2.68)	0.233	1.66 (0.87, 3.17)	0.123	No
		GG+AG	<15	1.84 (0.53, 6.42)	0.337	2.93 (0.78, 11.03)	0.112	No
		GG+AG	>25	1.87 (0.98, 3.57)	0.058	1.94 (0.98, 3.84)	0.059	No
rs1295686 (IL13 gene)	Dominant model	TC+CC	15 ~ 25	Ref.	–	Ref.	–	–
		TC+CC	<15	1.91 (0.54, 6.81)	0.319	2.88 (0.75, 11.01)	0.126	No
		TC+CC	>25	0.90 (0.45, 1.80)	0.765	0.98 (0.47, 2.03)	0.955	No
		TT	15 ~ 25	0.93 (0.53, 1.65)	0.796	1.09 (0.60, 1.97)	0.778	No
		**TT**	**<15**	**4.78 (1.20, 18.97)**	**0.027**	**5.96 (1.21, 29.43)**	**0.028**	**Yes**
		TT	>25	1.71 (0.96, 3.05)	0.068	1.65 (0.88, 3.07)	0.122	No
	Recessive model	CC	15 ~ 25	Ref.	–	Ref.	–	–
		CC	<15	2.07 (0.74, 5.85)	0.168	2.53 (0.86, 7.49)	0.092	No
		CC	>25	1.34 (0.85, 2.10)	0.208	1.21 (0.75, 1.96)	0.443	No
		TT+TC	15 ~ 25	0.61 (0.20, 1.82)	0.377	0.53 (0.17, 1.62)	0.265	No
		TT+TC	<15	1.68 (0.90, 3.14)	0.104	1.93 (0.99, 3.75)	0.054	No
		TT+TC	>25	1.27 (0.39, 4.08)	0.692	1.16 (0.29, 4.65)	0.833	No
rs20541 (IL13 gene)	Dominant model	AA+AG	15 ~ 25	Ref.	–	Ref.	–	–
		AA+AG	<15	0.67 (0.06, 8.06)	0.756	1.03 (0.10, 11.12)	0.979	No
		AA+AG	>25	2.56 (0.50, 13.22)	0.259	2.60 (0.43, 15.88)	0.293	No
		GG	15 ~ 25	2.23 (0.65, 7.65)	0.205	2.61 (0.74, 9.20)	0.137	No
		**GG**	**<15**	**4.55 (0.96, 21.56)**	**0.062**	**6.51 (1.30, 32.60)**	**0.025**	**Yes**
		GG	>25	2.95 (0.85, 10.25)	0.092	3.15 (0.88, 11.21)	0.076	No
	Recessive model	AA	15 ~ 25	1.00	–	1.00	–	–
		AA	<15	0.56 (0.11, 2.83)	0.506	0.90 (0.16, 5.16)	0.904	No
		AA	>25	0.88 (0.55, 1.40)	0.597	0.73 (0.45, 1.21)	0.219	No
		**GG+AG**	**15 ~ 25**	**0.58 (0.37, 0.93)**	**0.073**	**0.53 (0.33, 0.86)**	**0.022**	**Yes**
		GG+AG	<15	1.25 (0.42, 3.76)	0.457	1.38 (0.45, 4.23)	0.415	No
		GG+AG	>25	1.31 (0.79, 2.17)	0.105	1.29 (0.76, 2.20)	0.204	No
rs569108 (MS4A2 gene)	Dominant model	AA+AG	15 ~ 25	Ref.	–	Ref.	–	–
		AA+AG	<15	0.66 (0.21, 2.15)	0.414	0.82 (0.24, 2.78)	0.659	No
		AA+AG	>25	1.35 (0.88, 2.05)	0.185	1.27 (0.81, 1.99)	0.371	No
		**GG**	**15 ~ 25**	**0.59 (0.35, 0.99)**	**0.038**	**0.55 (0.32, 0.94)**	**0.016**	**Yes**
		GG	<15	2.43 (0.53, 11.13)	0.172	3.12 (0.66, 14.72)	0.095	No
		GG	>25	0.80 (0.45, 1.42)	0.404	0.68 (0.37, 1.25)	0.149	No
	Recessive model	AA	15 ~ 25	Ref.	–	Ref.	–	–
		AA	<15	1.21 (0.49, 2.97)	0.684	1.58 (0.62, 4.05)	0.336	No
		AA	>25	1.32 (0.92, 1.90)	0.136	1.24 (0.85, 1.81)	0.270	No
		GG+AG	15 ~ 25	1.06 (0.26, 4.32)	0.812	1.05 (0.26, 4.35)	0.766	No
		GG+AG	>25	1.59 (0.35, 7.23)	0.677	1.53 (0.33, 7.23)	0.760	No

Covariates adjusted for in multivariate analysis included season of birth, sex of the newborn, maternal allergy, household pet ownership, pre-pregnancy smoking, antibiotic use during pregnancy, birth weight, APGAR score, maternal BMI, maternal age.

Bold values: *p* < 0.05.

## 4 Discussion

This prospective birth cohort study provides novel insights into the complex relationship between early-life vitamin D exposure, genetic susceptibility, and childhood allergy development. As visualized in Figure 2, the U-shaped relationship between cord blood 25(OH)D and food allergy risk was statistically robust (*p* < 0.001 for non-linearity) during infancy, echoing prior reports of non-linear vitamin D effects on respiratory sensitization (32). However, our study extends this paradigm in three key dimensions: First, our U-shaped risk curve reflects dual genetic modulation: (i) risk amplification via Th2-pathway variants (IL4R/IL-4/IL13) under low vitamin D; (ii) protection attenuation in FcεRIβ (MS4A2) carriers at high vitamin D. Second, we establish this U-shaped relationship for clinically confirmed food allergy—a distinct phenotype from respiratory outcomes. Third, we reveal temporal dynamics: vitamin D’s protective effects attenuate by 24 months, suggesting critical developmental windows for intervention. While prior studies predominantly emphasize vitamin D deficiency as a risk amplifier for allergic disorders (5), our observation of heightened FA susceptibility at both low [25(OH)D <15 ng/mL] and elevated [25(OH)D >25 ng/mL] vitamin D levels challenges the linear “deficiency-risk” paradigm. This aligns with emerging evidence suggesting that excessive vitamin D may paradoxically impair immune tolerance by over-suppressing Th1/Th2 balance (16), though contrasting with reports advocating higher postnatal vitamin D supplementation for allergy prevention (20). Our observation of a U-shaped relationship between cord blood 25(OH)D and clinical food allergy contrasts with null findings for food sensitization in the Danish COPSAC2000 cohort (12), where vitamin D deficiency (<50 nmol/L [20 ng/mL]) only associated with respiratory outcomes. This discrepancy may reflect key methodological differences: (i) COPSAC assessed IgE sensitization rather than clinically confirmed food allergy; (ii) their cohort had substantially lower mean cord 25(OH)D (12.9 ng/mL) with minimal high-exposure representation (<1.5% > 30 ng/mL), potentially limiting detection of non-linear effects; and (iii) population-specific genetic/epigenetic factors may modulate vitamin D’s immunoregulatory effects. Importantly, our gene–environment interaction analyses suggest vitamin D’s impact is modified by genetic susceptibility, a dimension absent in prior null reports.

The gene–environment interactions we identified reveal a triad of Th2-sensitizing genotypes—IL4R rs1801275 AA, IL-4 rs2243250 CC, and IL13 rs20541 GG—that confer 4- to 26-fold elevated food allergy risk under vitamin D deficiency. This synergistic effect aligns with vitamin D’s role in suppressing IL-4/STAT6 signaling and Th2 differentiation via VDR-mediated chromatin remodeling (6). Our observation that deficiency overrides this suppression in specific genotypes suggests certain variants may create a “genetic vulnerability window” during fetal immune programming. This contrasts with studies where vitamin D supplementation mitigated allergy risk even in high-risk genotypes (6), highlighting the need for genotype-stratified intervention trials. Our results suggest that vitamin D sufficiency may be particularly critical for children with genetic variants predisposing to enhanced Th2 responses, potentially through restoration of immune balance between regulatory T cells and Th2 effectors. Unlike the uniformly risk-amplifying effects of Th2-pathway variants (IL4R/IL-4/IL13) under vitamin D deficiency, MS4A2’s role is protective and vitamin D-dependent.

The dual role of MS4A2 (rs569108) in vitamin D-dependent allergy modulation refines our understanding of genetic regulation in IgE-mediated responses. MS4A2 encodes the *β*-chain of the high-affinity IgE receptor (FcεRIβ) (23), a key amplifier of mast cell signaling. Contrary to initial hypotheses, we observed that the GG genotype conferred significant protection against allergy at reference vitamin D levels (15–25 ng/mL; adjusted OR = 0.55, *p* = 0.016), suggesting FcεRIβ may participate in vitamin D-mediated tolerance induction. However, this protective effect was attenuated at high vitamin D levels (>25 ng/mL; OR = 0.68, *p* = 0.149), indicating a therapeutic ceiling for supplementation in GG carriers. Notably, the absence of risk amplification under vitamin D deficiency (OR = 3.12, *p* = 0.095) diverges from Th2 gene interactions but aligns with vitamin D’s dose-dependent suppression of FcεRIβ expression (20). This complex gene–environment interplay underscores that genetic effects on IgE pathways are contextually modified by vitamin D status. Thus, while our U-shaped association aligns with broader evidence of vitamin D’s dual immunomodulatory roles, the discovery of genotype-dependent risk thresholds and age-specific susceptibility windows provides unprecedented mechanistic and translational insights for allergy prevention in genetically predisposed infants.

The transient nature of vitamin D’s protective effects—strongest at 6–12 months but diminishing by 24 months—echoes murine models where neonatal dendritic cell programming by vitamin D dictates long-term immune trajectories (9), yet diverges from birth cohort studies reporting persistent vitamin D-FA associations beyond infancy (12). The temporal pattern of vitamin D’s protective effects aligns with emerging concepts of developmental immunotoxicity windows. Experimental models demonstrate that vitamin D regulates neonatal dendritic cell function and gut barrier integrity during critical periods of microbial colonization (33). Our observation that vitamin D insufficiency during this period correlates with persistent food allergy risk underscores the importance of prenatal and perinatal vitamin D optimization, particularly given the rapid placental transfer of 25(OH)D during late gestation.

Notably, our finding of increased allergy risk at cord blood 25(OH)D levels >25 ng/mL warrants careful consideration. This aligns with findings from the Boston Birth Cohort, which indicated that persistently low vitamin D status at birth and in early childhood increased the risk of food sensitization, particularly among children with specific genetic susceptibilities (34). While the Institute of Medicine defines 20 ng/mL as sufficient for bone health (23), our data suggest potential immune system hypersensitivity to higher concentrations (35). The immunomodulatory effects of vitamin D are further illustrated by its impact on regulatory T cells (Tregs), which play a crucial role in maintaining immune tolerance (35). A study investigating the Urban Environment and Childhood Asthma cohort found that higher cord blood 25(OH)D concentrations were inversely related to the number of Tregs, suggesting that elevated vitamin D levels might reduce the capacity for immune regulation, thereby increasing allergy risk (36). This is supported by another study that found a negative correlation between cord blood 25(OH)D levels and Treg numbers, indicating that high vitamin D levels might impair the development of these critical immune cells (11). Such alterations in immune cell populations could contribute to the increased risk of food allergies observed at elevated vitamin D levels. Moreover, the role of vitamin D in allergy development is complicated by genetic factors. Polymorphisms affecting vitamin D-binding protein (DBP) can modify the relationship between serum vitamin D levels and food allergy risk. A study found that genetic variations leading to lower DBP levels, which increase the bioavailability of vitamin D, could attenuate the association between low serum vitamin D levels and food allergy, suggesting that genetic factors may influence how vitamin D impacts immune responses (37). Our observed U-shaped risk curve—particularly the vulnerability at high vitamin D levels—likely stems from genotype-dependent saturation of vitamin D receptor (VDR) signaling pathways, where excessive ligand promotes pathological immune remodeling in predisposed infants. This underscores that optimal vitamin D ranges for allergy prevention may be genotype-specific, with deficiency posing greater threat to Th2-sensitized infants. This genetic interplay highlights the complexity of vitamin D’s role in allergy development and suggests that individual genetic makeup can significantly alter the effects of vitamin D on immune function. However, the biological plausibility of this U-shaped association requires further investigation through mechanistic studies.

Several limitations should be acknowledged. First, while we adjusted for key covariates (e.g., maternal allergy history, smoking, and antibiotic use), residual confounding by unmeasured environmental factors may influence observed associations, especially maternal dietary patterns during pregnancy (38)—such as intake of allergenic foods (e.g., peanuts, eggs) or micronutrients (e.g., omega-3 fatty acids)—were not captured. Future studies should integrate dietary assessments to disentangle nutritional influences on fetal immune development. Second, the single-center design and ethnic homogeneity (Chinese Han population) may limit generalizability. Third, while cord blood 25(OH)D serves as a well-validated indicator of late-gestation fetal vitamin D exposure (25), we lacked maternal prenatal or infant postnatal vitamin D measurements. This precludes analysis of how maternal status independently influences allergy risk or whether postnatal supplementation modifies associations observed at birth. Future studies integrating longitudinal maternal–infant vitamin D trajectories would strengthen causal inference. Fourth, while our U-shaped association aligns with emerging evidence of vitamin D’s dose-dependent immune effects, it diverges from cohorts with distinct genetic backgrounds or environmental exposures. Generalizability to populations with severe vitamin D insufficiency (e.g., high-latitude cohorts) requires verification. Future multi-ethnic cohorts with repeated vitamin D measurements and detailed immune profiling could address these limitations.

## Data Availability

The data analyzed in this study was obtained from the Shanghai Allergy Cohort, the following restrictions apply: data sensitivity and institutional limitations. Requests to access these datasets should be directed to the corresponding author.
