# Supplementary material for: Association of cord blood vitamin D and genetic polymorphisms with childhood food allergy in Shanghai, China: a prospective cohort
Source: Front Nutr. 2025 Aug 25;12:1652487. doi: 10.3389/fnut.2025.1652487 (PMC12414776; doi:10.3389/fnut.2025.1652487)
Supplement: Supplementary file 1 [file Table_1.docx]

**Table S1. Associations Between Vitamin D Levels and Food Allergy: Univariate and Multivariate Analyses**

| **Allergy** | **25(OH)D level** | **Univariate analysis** | | **Multivariate analysis** | |
| --- | --- | --- | --- | --- | --- |
|  |  | **OR(95%CI)** | ***P* value** | **OR(95%CI)** | ***P* value** |
| 6-Month-Old Food Allergy | 15~25 | reference |  | reference |  |
|  | **<15** | **2.40(1.18,4.90)** | **0.016** | **2.55(1.20,5.43)** | **0.015** |
|  | **>25** | **2.56(1.38,4.72)** | **0.003** | **2.38(1.26,4.52)** | **0.008** |
| 12-Month-Old Food Allergy | 15~25 | reference |  | reference |  |
|  | **<15** | **2.08(1.35,3.19)** | **0.001** | **1.98(1.05,3.73)** | **0.035** |
|  | >25 | **1.62(1.12,2.36)** | **0.011** | 1.46(0.84,2.54) | 0.179 |
| 24-Month-Old Food Allergy | 15~25 | reference |  | reference |  |
|  | <15 | 1.55(1.05,2.28) | 0.027 | 1.14(0.45,2.89) | 0.779 |
|  | >25 | 1.05(0.75,1.47) | 0.785 | 1.26(0.62,2.57) | 0.530 |
| 0-24 month Overall Allergy | 15~25 | reference |  | reference |  |
|  | **<15** | 1.36 (0.90, 2.07) | 0.146 | **1.50 (0.99, 2.34)** | **0.044** |
|  | **>25** | 1.72 (1.18, 2.51) | **0.004** | **1.78 (1.19, 2.67)** | **0.005** |

**Table S2. Threshold Effect for 0-24 month Food Allergy**

| Outcome | | 6-Month-Old Food Allergy | 12-Month-Old Food Allergy | 24-Month-Old Food Allergy |
| --- | --- | --- | --- | --- |
| Cut-off point (K) | | 19.38 | 18.24 | 19.21 |
| Regression coefficients | **< Cut-off point** | 0.82 (0.72, 0.95) | 0.59 (0.36, 0.97) | 0.83 (0.73, 0.95) |
|  | **> Cut-off point** | 1.05 (1.01, 1.09) | 1.09 (1.00, 1.18) | 1.06 (1.02, 1.09) |

**Table S3. Analysis of the Interaction Between Genotype and 0-24 month Food Allergy**

| **Genetic Polymorphism** | **Genotype** | **Vitamin D Form** | **25(OH)D Level/Parameter** | **Food Allergy OR (95%CI)** | **P-value** |
| --- | --- | --- | --- | --- | --- |
| **IL4R rs1801275** | AA | Categorical | 15–25 ng/mL | Ref. | - |
|  |  | Categorical | **<15 ng/mL** | **4.13 (1.48–11.57)** | **0.007** |
|  |  | Categorical | >25 ng/mL | 1.26 (0.79–2.02) | 0.341 |
|  |  | Continuous (per 1 ng/mL) | - | **1.09 (1.02–1.17)** | **0.012** |
|  | AG | Categorical | 15–25 ng/mL | Ref. | - |
|  |  | Categorical | <15 ng/mL | 3.34 (0.96–11.65) | 0.058 |
|  |  | Categorical | >25 ng/mL | 1.28 (0.72–2.28) | 0.404 |
|  |  | Continuous (per 1 ng/mL) | - | 1.05 (0.98–1.13) | 0.165 |
|  | GG | Categorical | 15–25 ng/mL | Ref. | - |
|  |  | Categorical | <15 ng/mL | 4.30 (0.84–21.87) | 0.083 |
|  |  | Categorical | >25 ng/mL | 1.29 (0.64–2.60) | 0.477 |
|  |  | Continuous (per 1 ng/mL) | - | 1.03 (0.95–1.12) | 0.489 |
| **MS4A2 rs569108** | AA | Categorical | 15–25 ng/mL | Ref. | - |
|  |  | Categorical | <15 ng/mL | 1.58 (0.62-4.05) | 0.593 |
|  |  | Categorical | >25 ng/mL | 1.24 (0.85-1.81) | 0.944 |
|  |  | Continuous (per 1 ng/mL) | - | 1.03 (0.96-1.10) | 0.412 |
|  | AG | Categorical | 15–25 ng/mL | Ref. | - |
|  |  | Categorical | <15 ng/mL | 0.82 (0.24-2.78) | 0.659 |
|  |  | Categorical | >25 ng/mL | 1.27 (0.81-1.99) | 0.371 |
|  |  | Continuous (per 1 ng/mL) | - | 1.02 (0.95-1.09) | 0.587 |
|  | GG | Categorical | 15–25 ng/mL | Ref. | - |
|  |  | Categorical | <15 ng/mL | 3.12 (0.96-14.72) | 0.055 |
|  |  | Categorical | >25 ng/mL | 0.68 (0.37-1.25) | 0.149 |
|  |  | Continuous (per 1 ng/mL) | - | 0.95 (0.87-1.03) | 0.216 |
| **IL4 rs2243250** | TT | Categorical | 15–25 ng/mL | Ref. | - |
|  |  | Categorical | **<15 ng/mL** | **5.96 (1.21–29.43)** | **0.028** |
|  |  | Categorical | >25 ng/mL | 1.65 (0.88–3.07) | 0.122 |
|  |  | Continuous (per 1 ng/mL) | - | **1.14 (1.02–1.28)** | **0.021** |
|  | TC | Categorical | 15–25 ng/mL | Ref. | - |
|  |  | Categorical | <15 ng/mL | 2.88 (0.75–11.01) | 0.126 |
|  |  | Categorical | >25 ng/mL | 0.98 (0.47–2.03) | 0.955 |
|  |  | Continuous (per 1 ng/mL) | - | 1.02 (0.93–1.12) | 0.673 |
|  | CC | Categorical | 15–25 ng/mL | Ref. | - |
|  |  | Categorical | <15 ng/mL | 2.53 (0.86–7.49) | 0.092 |
|  |  | Categorical | >25 ng/mL | 1.21 (0.75–1.96) | 0.443 |
|  |  | Continuous (per 1 ng/mL) | - | 1.04 (0.95–1.14) | 0.418 |
| **IL13 rs1295686** | TT | Categorical | 15–25 ng/mL | Ref. | - |
|  |  | Categorical | **<15 ng/mL** | **6.51 (1.30–32.60)** | **0.025** |
|  |  | Categorical | >25 ng/mL | 3.15 (0.88–11.21) | 0.076 |
|  |  | Continuous (per 1 ng/mL) | - | **1.18 (1.03–1.35)** | **0.017** |
|  | TC | Categorical | 15–25 ng/mL | Ref. | - |
|  |  | Categorical | <15 ng/mL | 1.38 (0.45–4.23) | 0.415 |
|  |  | Categorical | >25 ng/mL | 1.29 (0.76–2.20) | 0.204 |
|  |  | Continuous (per 1 ng/mL) | - | 1.05 (0.96–1.15) | 0.286 |
|  | CC | Categorical | 15–25 ng/mL | Ref. | - |
|  |  | Categorical | <15 ng/mL | 0.90 (0.16–5.16) | 0.904 |
|  |  | Categorical | >25 ng/mL | 0.73 (0.45–1.21) | 0.219 |
|  |  | Continuous (per 1 ng/mL) | - | 0.97 (0.89–1.06) | 0.512 |
| **IL13 rs20541** | GG | Categorical | 15–25 ng/mL | Ref. | - |
|  |  | Categorical | <15 ng/mL | 3.12 (0.66–14.72) | 0.095 |
|  |  | Categorical | >25 ng/mL | 0.68 (0.37–1.25) | 0.149 |
|  |  | Continuous (per 1 ng/mL) | - | 0.96 (0.88–1.05) | 0.347 |
|  | AG | Categorical | 15–25 ng/mL | Ref. | - |
|  |  | Categorical | <15 ng/mL | 0.82 (0.24–2.78) | 0.659 |
|  |  | Categorical | >25 ng/mL | 1.27 (0.81–1.99) | 0.371 |
|  |  | Continuous (per 1 ng/mL) | - | 1.03 (0.96–1.11) | 0.428 |
|  | AA | Categorical | 15–25 ng/mL | Ref. | - |
|  |  | Categorical | <15 ng/mL | 1.58 (0.62–4.05) | 0.336 |
|  |  | Categorical | >25 ng/mL | 1.24 (0.85–1.81) | 0.27 |
|  |  | Continuous (per 1 ng/mL) | - | 1.02 (0.95–1.10) | 0.593 |

* Covariates adjusted include Season of Birth, Sex of the Newborn, Maternal Allergy, Household Pet Ownership, Pre-pregnancy Smoking, Antibiotic Use During Pregnancy, Birth Weight, APGAR Score, Maternal BMI, Maternal Age.
Odds ratios (OR) and 95% confidence intervals (CI) were calculated using logistic regression models, clearly demonstrating the interaction effect between vitamin D and food allergy risk across different genetic backgrounds.
